# BUILD EXITO: a multi-level intervention to support diversity in health-focused research

**DOI:** 10.1186/s12919-017-0080-y

**Published:** 2017-12-04

**Authors:** Dawn M. Richardson, Thomas E. Keller, De’ Sha S. Wolf, Adrienne Zell, Cynthia Morris, Carlos J. Crespo

**Affiliations:** 1 School of Community Health, College of Urban and Public Affairs, Portland State University, Portland, OR 97207 USA; 2 School of Social Work, Portland State University, Portland, OR 97207 USA; 3 Center for Interdisciplinary Mentoring Research, Portland State University, Portland, OR 97207 USA; 4 Oregon Clinical and Translational Research Institute, Oregon Health & Science University, Portland, OR 97239 USA

## Abstract

**Background and purpose:**

As part of the NIH BUILD initiative to diversify the scientific workforce, the EXITO project is a large multi-institutional effort to provide comprehensive support and training for undergraduates from traditionally underrepresented student populations who aspire to health-related research careers. Portland State University, a major public urban university that prioritizes student access and opportunity, and Oregon Health & Science University, a research-intensive academic health center, lead the EXITO network comprised of eleven 2-year and 4-year institutions of higher education spanning Oregon, Washington, Alaska, Hawaii, Guam, American Samoa, and the Northern Mariana Islands. The EXITO project aims for impact in biomedical research by training diverse scholars from indigenous and underserved communities affected by adverse health disparities.

**Project approach:**

Guided by socio-ecological theory, the EXITO project is a multi-level intervention offering a three-year research training pathway for scholars in the biomedical, behavioral, health, and social sciences. Fundamental components of the model include student outreach and engagement, integrated curricular enhancements, intensive research experiences, multi-faceted developmental mentoring, supportive community and services, and rigorous evaluation and quality improvement. EXITO also advances faculty and institutional development in these domains by holding curriculum development conferences, creating research learning communities, awarding pilot project research funding, providing mentor training and ongoing support, collaborating with other research equity programs, and developing campus infrastructure and services to support scholars with diverse backgrounds and needs.

**Highlights:**

The large and geographically broad network of EXITO institutions engages a range of diverse students, including indigenous populations and students beginning post-secondary education at community colleges. The EXITO model specifically accommodates many students transferring from 2-year partner institutions and facilitates seamless transfer to the 4-year institution. EXITO features several approaches to research training, including supported summer entry into research placements, the incorporation of responsible conduct of research content into general education curriculum, and the intentional matching of scholars with three types of mentors (e.g., peer, career, research).

**Implications:**

EXITO provides an example of a comprehensive research training initiative for traditionally underrepresented students that can be implemented across a diverse range of 2-year and 4-year institutions.

## Background

The U.S. faces staggering racial, ethnic, and socioeconomic health disparities. As population demographics shift, it is critical that the health research workforce responds with innovative approaches to address and eliminate health disparities. One key strategy is to promote the scientific training of individuals who reflect and represent the diverse communities most affected by adverse health outcomes [1, 2]. Currently the biomedical research workforce does not mirror the diversity of the U.S. population [3–5]. Recognizing the need to address the issue of underrepresentation in the social, biomedical, behavioral, and clinical sciences [4], the National Institutes of Health (NIH) established the Building University Infrastructure Leading to Diversity (BUILD) initiative with ten grantees nationwide [6]. Portland State University (PSU) was selected as a BUILD site to develop and implement EXITO (‘Enhancing Cross-disciplinary Infrastructure and Training at Oregon’), a project aimed at learning how to effectively recruit, retain, support, and train diverse undergraduate students to develop the skills and capacities needed to become NIH-fundable researchers.

Students from traditionally underrepresented backgrounds face distinctive challenges in the pursuit of higher education. Low-income, first-generation students, for example, encounter a number of unique barriers: they are less likely to receive financial support from parents, resulting in part-time enrollment while working to pay tuition; they are more likely to have family and work obligations, which slows progress towards degree completion; and more likely being from racial and ethnic minority backgrounds, which have lower rates of college engagement so these students often receive less social support, experience discrimination, and have trouble connecting with faculty and mentors from similar backgrounds [7–10]. Retention of students in STEM (Science, Technology, Engineering, and Math) majors is also a challenge [11], with women, underrepresented minorities, low-income, and first-generation students experiencing particularly low enrollment and high attrition rates [12–15]. In addition to the barriers described above, there is evidence that student attitudes (e.g., motivation, confidence, science identity, self-efficacy for STEM learning) [13, 15] as well as institutional characteristics (e.g., emphasis on teaching versus research) [14] play a role in disparate attrition rates.

**Fig. 1:**
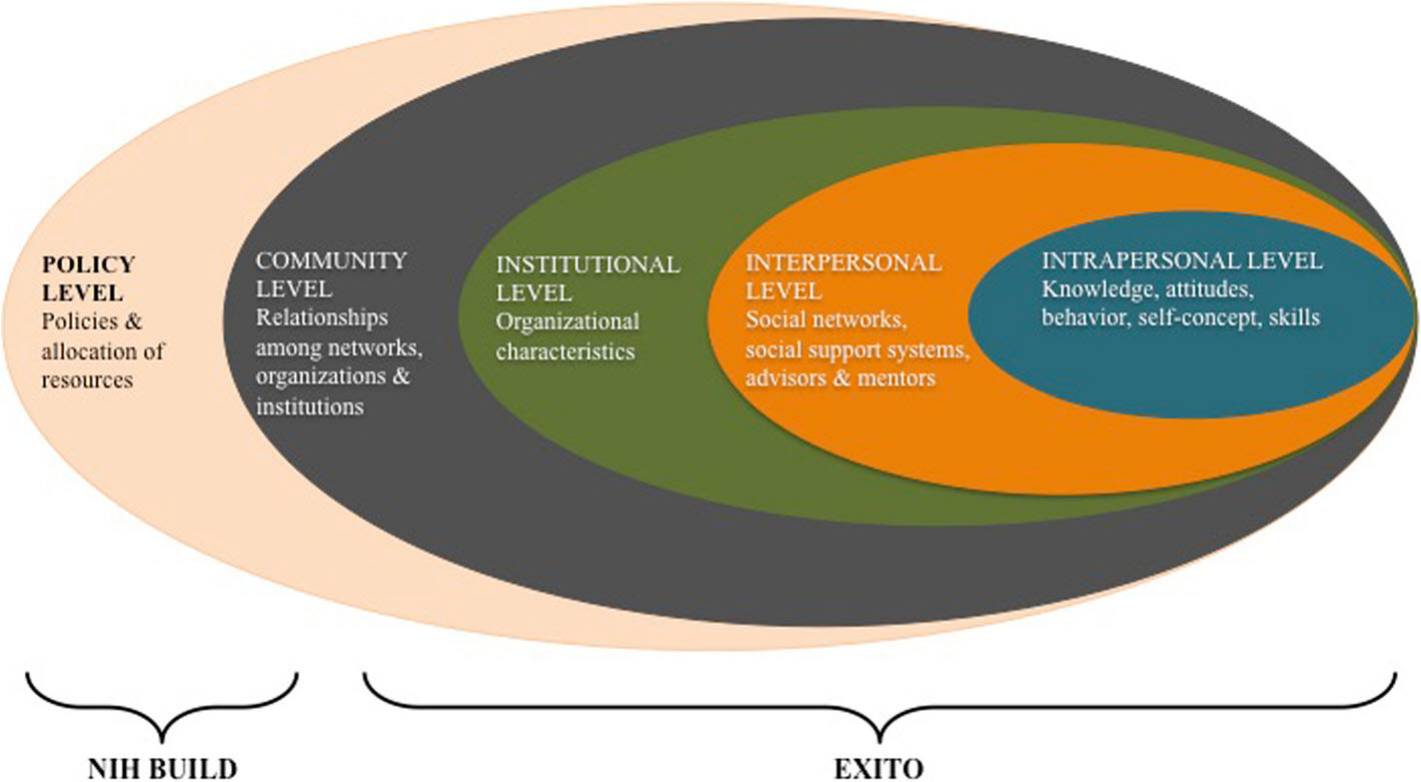
BUILD EXITO Conceptual Framework: Levels of Intervention

Building on established theoretical work emphasizing the importance of student context [16, 17], the EXITO project is shaped by socio-ecological theory [18], a conceptual framework with roots in education theory [19]. Socio-ecological theory positions individuals within nested environmental contexts, facilitating consideration of multiple intervention opportunities. This conceptual framework suggests that individual behavior and experiences are shaped and reified by factors across a spectrum including the intrapersonal, interpersonal, institutional, and community levels (see Fig. 1). Socio-ecological theory is congruent with the overarching goals and structure of the BUILD initiative, which highlights the need to implement change on multiple levels (student, faculty, institution) to educate and empower undergraduates from traditionally underrepresented student populations who aspire to careers in health-focused research. Using this framework as a guide, as well as more recent adaptations of established conceptual models [20], the EXITO project has incorporated longstanding theory on recruiting and retaining underrepresented students in higher education [21–25]. Further, because the socio-ecological framework stresses intervention points across a spectrum, it encourages the development of strategies that complement and reinforce each other across multiple levels of influence to achieve sustainable outcomes.

Within the broader policy context framed by NIH and the BUILD initiative, the EXITO project strives to be holistic in combining specific intervention components across levels of the socio-ecological framework. For example, EXITO aims to transform the individual educational experiences of participating students by providing curricular enhancements, research opportunities, multi-faceted mentoring, and supportive services that promote their academic success, research preparation, career planning, and psychosocial development. However, in addition to implementing innovative research training, the EXITO project focuses on building the institutional capacity and infrastructure needed to support and sustain a comprehensive research-focused diversity initiative. Specifically, EXITO includes faculty development efforts that promote curricular innovations incorporating research experiences into courses, create opportunities for faculty to engage in research and pursue external funding, and provide training and support on effectively mentoring undergraduate students from diverse backgrounds. Additionally, the EXITO project extends the “biomedical” umbrella to include clinical, social, and behavioral sciences because the causes and consequences of health disparities are complex and multifaceted, so efforts to reduce and eliminate these disparities require input from a range of disciplines.

This article presents program components by intervention level, demonstrating how the EXITO project aims to meet the complementary, mutually reinforcing goals of student training and institutional development. Each section includes a figure depicting how we believe change will be achieved. We then share observations on the challenges and opportunities associated with undertaking a complex, multi-institutional initiative to create an academic pathway for students from diverse backgrounds to pursue careers in biomedical and social science careers. The article concludes with an overview of our evaluation and its alignment with the BUILD hallmarks of success.

## Community level: EXITO partnership network

**Fig. 2:**
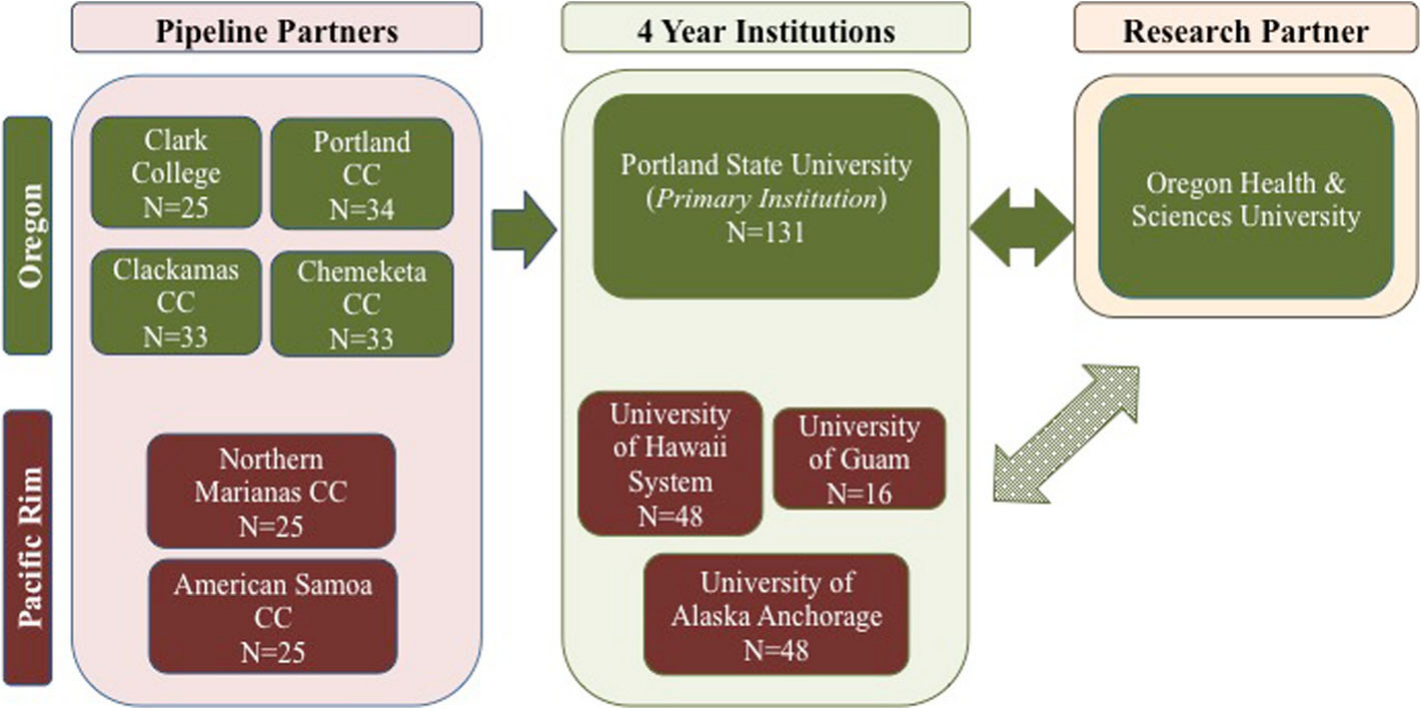
BUILD EXITO Partnership Network

The community-level context of the EXITO project is defined as the geographically diverse partnership network of eleven institutions spanning four states, three territories, and eight time zones across the U.S. Pacific region (see Fig. 2). The institutional structure of the BUILD program (as initially conceptualized by NIH) specifies a primary institution (PSU), a research partner (OHSU), and pipeline partners (2-year community colleges), each with a principal investigator (PI) from its own faculty. In addition to these participants, the EXITO project network includes 4-year university partners. Spearheaded by the primary grantee institution, PSU, the development of the EXITO network was driven by existing collaborations with the research-intensive institution (OHSU), established student enrollment pathways (local community colleges), and intentional efforts to foster new relationships with strategic partners (Pacific Rim partners).

Two student pathways are represented in the partnership network: students can begin their EXITO training at a two-year partner institution and then transfer to PSU for the remainder of their training; alternatively, students already enrolled at a four-year partner institution receive the full EXITO training experience on their home campus. Thus, the emphasis with two-year partner institutions is effective alignment and articulation of respective program components at each developmental level, whereas the emphasis with four-year partner institutions is successful replication of all program components. As the primary institution in this spoke and hub network, PSU provides leadership and centralized staffing for core program development and administrative functions. The partner institutions have flexibility to adapt the standard program models and administrative procedures for implementation with reference to their own contexts and capacities. Given the interdependent relationships between institutions, effective communication and efficient coordination are critical. EXITO project efforts at the community level include: the development of collaborative, multi-site working groups; monthly partner meetings with video conferencing technology to facilitate engagement across a range of time zones; and utilization of online platforms to share curriculum and student support resources.

### Oregon partners

#### Primary institution: Portland State University

The primary institution awarded BUILD funding for the EXITO project is Portland State University [26]. PSU is Oregon’s largest and most diverse university***,*** with students, faculty, and staff from over one hundred countries and all fifty states, representing a broad spectrum of religions, ethnicities, tribes, sexualities, genders, abilities, ages, identities, and experiences. As the only major urban university in Oregon, PSU has an established history of providing educational opportunity to students from diverse backgrounds who otherwise may not have access to public higher education. PSU serves a large population of first-generation college students, low-income students, and transfer students. In one of the most recent cohorts of new undergraduate students (2015), 33% self-identified as members of a racial or ethnic minority group, and 64% of first time enrollees were transfer students (rather than first-time freshmen). PSU offers a range of undergraduate degrees that prepare students for health and biomedical research, including majors in biology, chemistry, community health, engineering, environmental science, psychology, sociology, and social work. The institutional home for EXITO at PSU is the Center for Interdisciplinary Mentoring Research (CIMR), established in 2010 to enhance mentoring across the lifespan via research, education and knowledge transfer, and partnerships with organizations providing services. As the largest program site and lead institution, PSU has core project staffing that includes faculty leadership of administrative, institutional development, student training, and research enrichment cores as well as project-wide functional positions such as project manager, academic adviser, communications manager, operations coordinator, and project coordinators.

#### Community college pipeline partners

The local EXITO institutional network includes four community colleges that contribute a large number of transfer students to PSU - Chemeketa Community College (Salem, OR), Clackamas Community College (Oregon City, OR), Clark College (Vancouver, WA), and Portland Community College (Portland, OR). These 2-year institutions are primary providers of public higher education in the Portland metropolitan area and serve diverse student populations, with particularly high representation of Latino students. Capitalizing on PSU’s established partnerships with these community colleges, EXITO leverages dual enrollment agreements allowing students to matriculate in both institutions after completing just one common application process. Through dual enrollment, students gain greater flexibility in course scheduling, with access to classes at either institution. Students also have access to services and opportunities on both campuses. At each pipeline partner, a faculty member within a biomedical discipline serves as the Principal Investigator for the respective EXITO subcontract and typically works with additional faculty colleagues to manage program functions (e.g., student recruitment) and program components (e.g., research gateway course).

#### Research-intensive partner: Oregon Health & Science University

The research-intensive partner in the EXITO project is Oregon Health & Science University (OHSU), a comprehensive academic health center that features patient care, medical education, and an extensive world-class research portfolio. OHSU, Oregon’s only academic medical center, has graduate schools of medicine, nursing, dentistry, pharmacy, and public health. With approximately $375 million in annual research expenditures, more than 1500 OHSU scientists work on over 4000 basic, clinical, translational, and applied research projects. A multi-layered partnership exists between OHSU and PSU, facilitated by close geographic proximity, leadership committed to collaboration, and several initiatives such as development of a joint School of Public Health and construction of a Collaborative Life Sciences Building that co-locates researchers, educators and students from both institutions. Although OHSU does not enroll undergraduates, it has a long history of training and outreach programs providing enriched educational opportunities for undergraduates, particularly those from traditionally underrepresented populations. As described below, faculty researchers at OHSU offer research placement opportunities for EXITO scholars.

### Pacific-rim partners

The EXITO partnership network connects PSU and OHSU with institutions in Alaska, Hawaii, and the U.S. Pacific Island territories of American Samoa, Guam, and Northern Mariana Islands. EXITO forged these new relationships to engage students that historically have had limited access to health science and biomedical training opportunities due to their isolated locations, particularly American Indian/Native Alaskan, Native Hawaiian, and Pacific Islander populations. The Pacific partners include American Samoa Community College and The Northern Mariana College (2-year colleges), as well as the University of Alaska Anchorage and University of Guam, which are both 4-year universities. An additional EXITO partner is the statewide University of Hawaii system, comprised of seven community colleges and three universities, including a major research university with a medical school. University of Hawaii Manoa serves as the primary EXITO connection with the Hawaii system, and the project is well situated in the Department of Native Hawaiian Health. Collectively, these partner institutions enroll large numbers of students from traditionally underserved communities that bear a heavy burden of health disparities. In addition, they enrich collaboration within the EXITO network through their expertise in native and indigenous health. As at other partner institutions, faculty in biomedical disciplines serve as PIs for the EXITO subcontract and manage local program operations.

## Institutional level: Institutional development

**Fig. 3:**
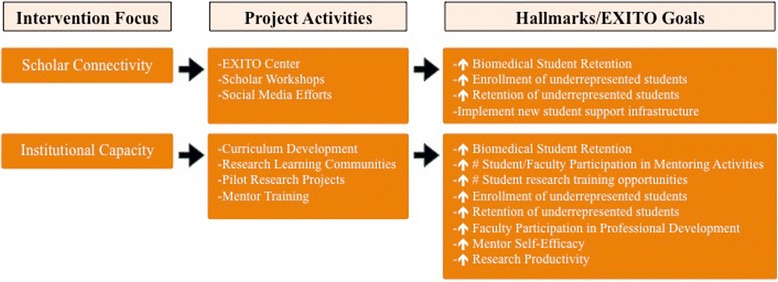
BUILD EXITO Conceptual Framework: Change at the Institutional Level

While the EXITO project is developed and coordinated by the partnership network at the community level, students intersect with the project at the institutional level; therefore, EXITO universities and colleges are critical points of intervention. Social integration into the campus setting is important for enhancing student persistence [24], so creating institutional environments that are accessible and supportive of students is critical for project success. Promoting a diverse, welcoming campus climate is of particular importance for underrepresented student success and retention [7, 17, 27, 28], and by creating and sustaining these conditions academic institutions can optimize student outcomes [17, 29]. Thus, to meet the BUILD hallmarks of success and EXITO project goals, institution-level changes to enhance student support are prioritized (see Fig. 3). These efforts include the development of concrete strategies for engaging and supporting diverse students, facilitating student connections to each other [29, 30], the institutionalization of these processes, and corresponding efforts to build institutional capacity across our partnership [29, 31].

### Institutionalizing student connections

#### EXITO center

A concrete example of institutional development to promote a supportive environment for scholars is the EXITO Center, a physical space created as a “home base” on the PSU campus. The EXITO Center serves as a hub connecting students to project staff and advisers, opportunities to work with peers, and other activities and events that support learning and foster a sense of shared purpose and community. The Center features community-learning spaces to host speakers, seminars, workshops, gatherings, and group project meetings, and also includes office space for EXITO Academic Advisers and project staff. In addition, the EXITO Center space is co-located with other mentoring and support programs with a focus on student diversity and science education, including McNair Scholars [32], Louis Stokes Alliance for Minority Participation (LSAMP) [33], and the STEM Institute [34]. Bringing these programs together under one roof facilitates productive exchange and collaboration to promote greater quality and efficiency of services. It also facilitates important interactions among the diverse students they serve.

#### EXITO scholar workshops

Across the partnership a number of workshops and seminars have been developed in collaboration with other campus programs to facilitate the delivery of EXITO curriculum. By actively involving representatives from campus resource centers, such as multicultural, disability, advising, career services, and academic support centers, the EXITO project is institutionalizing collaborative efforts and helping to build cohorts of diverse, underrepresented students across programs and across campus. Building on and extending these existing institutional resources, the EXITO project is better positioned for sustainability, and EXITO Scholars are supported in building needed networks.

#### Social media efforts

Implementation of the EXITO student engagement activities has coincided with the development of institutional infrastructure to support these efforts. A variety of communications platforms and products (e.g., social media channels, websites, videos, online application portal) have been established for use by all EXITO partners for outreach and recruitment, as well as continued scholar engagement.

### Building institutional capacity

EXITO institutional development activities focus most intensely on building, enhancing, and institutionalizing the capacity of PSU and partner institutions to develop and implement science-based curriculum and to offer sustainable research training opportunities for diverse scholars.

#### Curriculum development

To foster the development and adoption of novel science instruction approaches, EXITO faculty from all partner institutions convene for an annual summer Curriculum Development Conference. During this week long meeting, faculty share strategies and pedagogical approaches for enhancing science-oriented curriculum. For example, the first conference was facilitated by experts from PSU’s Office of Academic Innovation and featured sessions by faculty working on a Howard Hughes Medical Institute grant to incorporate deliberative democracy discussions into science courses. Each conference also addresses the continuing development and refinement of the common curriculum required of all EXITO scholars prior to beginning their research placements. The Gateway course (further described in under ‘EXITO Curriculum’) is a foundational research course that includes content on scientific inquiry, research methodology, and the responsible conduct of research. With support from the EXITO project, this course is being institutionalized on all partner campuses, facilitating receipt of course credit for transfer students.

#### Research learning communities

Given the importance of team science in the production of high-impact findings [35], the EXITO project has made the Research Learning Community (RLC) [36] a pivotal capacity building strategy. RLCs are collaborative research teams that vary in structure and composition, typically including an established principal investigator (i.e., with major federal grants) as well as faculty co-investigators, early-career investigators, fellows, post-docs, and graduate students. This multi-level membership supports a tiered mentoring approach in which senior researchers guide and sponsor early career faculty, positioning them to secure extramural funding. RLC members receive training emphasizing the promotion of a sustainable culture of mentorship, inclusion, and diversity within research teams, including a focus on the learning experiences of researchers from diverse backgrounds. To support the expansion of research initiatives, each RLC is eligible for an annual installment of research stimulus funding to be devoted toward professional development or research development expenditures. In this way RLCs are a mechanism for enhancing institutional research capacity by supporting faculty development and fostering collaborations that generate new projects and proposals.

#### Pilot research projects

EXITO Pilot Research Projects (PRP) [37] replicates the NIH R03 mechanism (both the application and review process) to provide competitively awarded seed funding ($50,000) for new research projects that also engage EXITO Scholars. The RFA is open to faculty across the EXITO partnership and calls for applicants to submit all materials required for an R03 proposal plus a mentoring plan that should describe how the project will (a) incorporate research-training strategies for EXITO scholars, and (b) support early-career researchers in preparing for biomedical research careers. Applicants are connected with the faculty and career development services available through OCTRI and grant-writing workshops offered by the National Research Mentoring Network (NRMN) [38]. The principal investigators of funded pilot projects meet quarterly for progress updates on their research and coaching on manuscript and grant preparation from an experienced writer and editor. Through the pilot projects, faculty and EXITO scholars gain insight into the process of proposal preparation and review; even unsuccessful applicants receive the benefit of receiving high quality reviews, building their capacity for pursuing additional external funding opportunities.

#### Mentor training

EXITO institutional development includes extensive training for faculty and students serving as mentors (including and extending beyond core EXITO faculty and scholars). The core content for this professional development is based on the interactive modules of the *Entering Mentoring* curriculum developed by leading experts associated with NRMN [39]. Mentors learn to provide culturally sensitive support for scholars and other students through modules addressing equity and inclusion, aligning mentor/mentee expectations, building communication skills, and supporting career development, among other important topics. Institutional development included building the capacity for each institution to deliver this mentor training curriculum by having EXITO faculty representatives participate in NRMN’s train-the-trainer certification process.

## Interpersonal level: Mentoring & supporting EXITO scholars

**Fig. 4:**
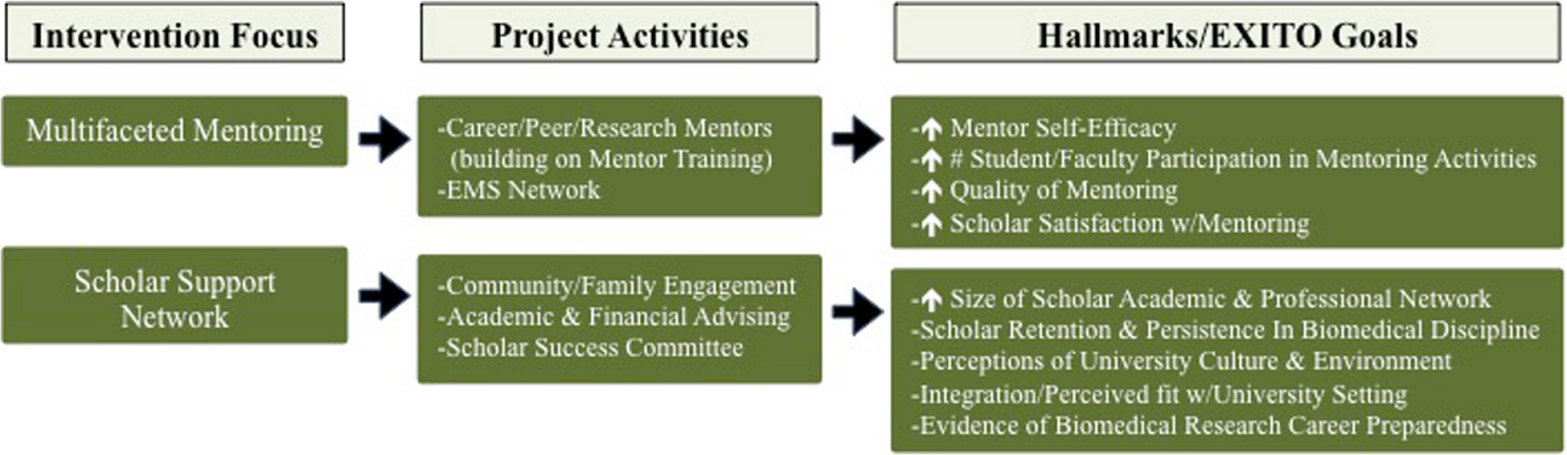
BUILD EXITO Conceptual Framework & Evaluation: Interpersonal Level

The interpersonal level comprises the system of social influence and support from peers, family members, and other key actors in an individual’s network, as well as the norms and practices shaping this system. Integrated support services are critical for students success [30]. At the interpersonal level, the EXITO project focuses on providing mentoring as well as instrumental assistance to meet scholar needs. Mentorship is an important strategy for promoting student success [40], and following guidance on best practices [41], EXITO scholars receive tiered mentoring across multiple levels, with all mentors (career, peer, research) receiving training [42]. Recognizing that the provision of trained, well-matched mentors is not sufficient [43, 44], each mentor/mentee relationship receives ongoing monitoring and support from an EXITO project coordinator. In addition to this multiple mentor model, EXITO provides scholars with a range of advising services and a network of social support [42], including a plan for engaging families [29]. We believe this provision of structured support will achieve project goals and facilitate our ability to meet the overarching BUILD Hallmarks of Success (see Fig. 4).

### Multifaceted mentoring

Recognizing the multi-dimensional needs of students from traditionally underrepresented backgrounds embarking on a demanding research-oriented academic career [45], EXITO employs a team mentoring approach (see Keller et al. for a comprehensive descriptions of the EXITO mentoring model). As described below, there are three distinct mentoring roles in the EXITO model. Each type of mentor reflects a different perspective, has a different set of priorities, and offers a different mix of skills and support.

#### Career mentors

Each EXITO scholar is matched to a career mentor, a faculty member with a research background. Career mentors receive training to work in an effective and culturally competent manner with EXITO scholars. As the scholar’s enduring “champion” or “sponsor,” the career mentor provides advice, guidance, encouragement, and support to EXITO scholars on a range of topics including navigating higher education, gaining research experience, choosing a career path, developing a strong CV, writing proposals and manuscripts, applying to graduate programs, networking and making connections. To promote institutional development through mentorship and mentor training, we have adopted an intentionally broad reach for identifying, recruiting, and selecting career mentors.

#### Peer mentors

The EXITO peer mentoring program employs the “near-peer” approach, in which a student is matched with a mentor who shares a similar background but already has navigated a pathway to the type of success desired by the mentee. Such a mentor is likely to have strong credibility with the scholar, as the mentor recently achieved a goal similar to that of the mentee and understands what is involved in making it a reality [41]. EXITO peer mentors serve as guides to student life and success, helping EXITO Scholars connect to campus cultural activities, groups, and programs as well as navigate university services such as housing, financial aid, and recreation. Additionally, EXITO peer mentors share personal insights and counsel scholars on how to take advantage of EXITO courses, resources, and research experiences.

#### Research mentors

Mentorship is central to undergraduate research; in the EXITO project this occurs in the context of the RLCs, which feature multiple investigators at various stages of career development. These mentor-rich environments provide opportunities for tiered, team mentoring of EXITO scholars by scientists with different levels and varying expertise [41]. Each scholar in an RLC must be designated a primary research mentor who essentially serves in a supervisory role, providing the scholar with day-to-day training, consultation, and guidance on both practical and conceptual issues related to the research. In this way, the research mentor is responsible for the continuing growth and development of the scholar with increasing levels of challenge and responsibility within the position over time.

#### EXITO mentoring support network

Because the EXITO mentoring program spans multiple partnering institutions in geographically diverse locations, it employs an innovative online platform- the EXITO Mentoring Support Network (EMSN)- as infrastructure for communication with all mentors and scholars [43]. Mentors and scholars have individual accounts and receive regularly scheduled email prompts to respond to questions within the system. Certain consistent questions elicit information about the nature and development of the mentoring relationship, while other questions are customized to inform project improvement. EMSN also provides a forum for announcements and sharing resources from the program and among participants. In addition, EMSN compiles and analyzes data across the mentoring relationships for tracking and reporting purposes.

### Scholar support network

The EXITO project attempts to address the social, academic, and financial needs of scholars from backgrounds that have traditionally encountered institutional barriers. These efforts include an intentional focus on network-building, enhanced and dedicated advising, and attempts to coordinate seamless scholar support.

#### Building support networks

Feeling connected to a learning community fosters a sense of belonging and identity [46], so EXITO provides intentional, structured opportunities for students to gather, learn from, and support one another. Scholars are connected with a variety of campus opportunities and services, including research fairs, student groups, and cultural centers. EXITO includes a family engagement plan for scholars to develop strategies for securing family support of academic and career goals. Across the partnership a working group has been convened to continue to identify unique challenges and opportunities for network-building and family engagement, and to develop a core set of engagement principles to be adapted by each site.

#### Academic & financial advising

First generation college students and students from underrepresented backgrounds face unique challenges navigating their undergraduate educations, so EXITO employs a full-time Academic Advisor and commits .25 FTE to a dedicated Financial Aid Advisor. These advisors promote understanding of degree and program requirements, scholarships and financial aid, university policies and procedures, and the student transfer process. Among the many strategies used to support scholars include: (a) required academic advising appointments once per term; (b) required academic plans; (c) a transfer planning guide; (d) outreach emails notifying Scholars of registration dates, scholarship opportunities, and important academic deadlines; and (e) individual development plans.

#### Scholar success committee

The EXITO program sets high expectations for academic achievement and program engagement and holds scholars accountable for completing program milestones. Scholar Agreements outline the support and training EXITO will provide along with expectations for scholar GPA, research hours, required advising, and timely communication with the program, especially when challenges occur. Many cases that come to the student success committee are the result of scholar crises involving finances, housing, medical issues, child care, or other family concerns. In these instances, the Student Success Committee engages in problem-solving approaches with the scholar to stabilize the situation and maintain program participation, often drawing on campus resources or making referrals to campus or community services.

## Intrapersonal level: Curriculum & training

At the intrapersonal level, the EXITO project focuses on the knowledge, attitudes, and beliefs of scholars. To foster success at this level, EXITO provides scholars with enhanced science curriculum, skills-building workshops and seminars, and research training opportunities. These program components are delivered as an integrated set of experiences along a three-year pathway from the end of freshman year to graduation (see Table 1 for the sequence of major program activities).

**Table 1:** Research Training Timeline for EXITO Scholars

Year	Timing^a^	Program component	Purpose
1 (Freshman)	Fall	Enhanced introductory science and general education courses	Provide positive initial exposure to health/science education for all students
	Spring	Scholar recruitment/admission process	Select eligible students with high motivation and potential
	Summer	Scholar orientation (1 week)	Orient to program, build cohort, begin developing science identity and goals
		Academic advising (ongoing)	Map course expectations, discuss credit transfers, other planning
2 (Sophomore)	Fall	Career mentor match (ongoing)	Offer general support and advice from faculty member
		Peer mentor match (ongoing)	Offer general support and advice from experienced student
		Enrichment workshops (ongoing)	Build cohort, provide relevant information and training (personal, social, academic)
		Gateway course	Provide basic research foundation—scientific principles and responsible conduct of research—prior to placement with RLC
	Spring	Gateway course (if not done in Fall)	See above
	Summer	Summer Induction experience (1-month)^ab^	Provide supported entry into RLC; workshops reinforce Gateway content
3 (Junior)	Fall	RLC placement (ongoing)	Offer practical training/experience working on faculty-driven research project
		Research mentor match (ongoing)	Offer supervision and training on research procedures relevant for RLC project
	Summer	Summer Immersion experience (3-month)	Permit continuation of paid work in RLC; provide professional development support
4 (Senior)	Fall	Scholar-driven research (capstone)	Support demonstration of skills developing and conducting research
		Application to graduate school	Provide supports for selecting and applying to graduate programs
	Spring	Graduation	Continue trajectory to graduate education

^a^Timeline indicates when program components are introduced, many of which are ongoing

^b^Summer induction can serve as a bridge for transfer students entering a 4-year partner institution at this point

**Fig. 5:**
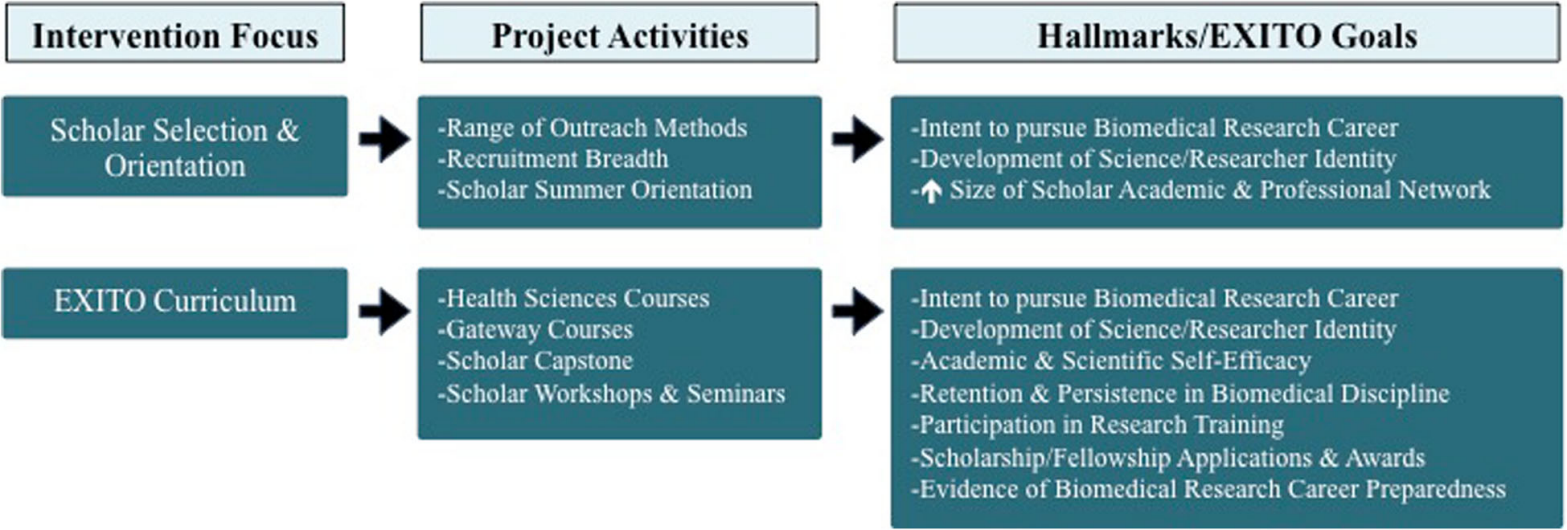
Research Preparedness Model

These activities are meant to build scholar science identity and research preparedness, both of which are linked to pursuit of postgraduate training and science careers [5, 47–49]. The benefits of research training for undergraduates are well-documented [50–56], with recent studies highlighting how they build research preparedness among undergraduates [57–59]. These benefits are especially significant for traditionally underrepresented students, both women and racial/ethnic minorities, and particularly for those lacking adequate support systems, as these students tend to rate themselves lower on research self-efficacy [58]. Consequently, these students benefit most from preparatory work prior to engagement in research experiences and, ultimately, from research participation in terms of improved GPA, retention in STEM degree programs, and pursuit of science courses [57]. In order achieve project aims as well as the BUILD hallmarks of success, the EXITO project curriculum and research training are designed to address these components of preparedness while ensuring that scholars receive a continuum of multi-dimensional support and development opportunities (see Fig. 5).

### Scholar selection & orientation

#### Outreach and recruitment

Each EXITO partner (with the exception of OHSU, which does not serve undergraduate students) recruits, selects, and enrolls a new cohort of EXITO scholars who are in the spring of their first year in college. Reflecting the range of the NIH, the EXITO project is broad and inclusive with respect to the scientific interests of students and recruits undergraduates from a wide range of majors. Based on criteria established by NIH, eligible students are from traditionally underrepresented populations based on (a) racial or ethnic minority status; (b) severe social or economic disadvantage; or (c) experiences of disability (see Table 2). Recruitment strategies include social media campaigns, announcements shared via email and websites, classroom visits, and word-of-mouth referrals. Applicants undergo a competitive selection process that involves completion of an online application and interviews with project staff. To support prospective applicants, EXITO offers in-person workshops, online tutorials, and peer mentor outreach to provide individual coaching. Selected students are designated as EXITO scholars. Scholars selected at 2-year partner institutions participate in EXITO program activities at their own community college for one year and then transfer to a partner 4-year institution, where they continue for the final two years of the EXITO program. Scholars selected at 4-year partner institutions complete the EXITO program activities at their home university.

**Table 2:** Selection Criteria for EXITO Scholars

Phase	Assessment Criteria	Evaluation
Eligibility	Established by NIH: Citizenship status; Enrollment status	PSU Evaluation Committee
Application Review	GPA (2.5 minimum)	Scoring^a^ by Site Evaluation Committee, 0–2 scale. 3 reviewers per applicant; those receiving divergent scores (2-point difference on 2+ criteria) reviewed by 4th member.
	Exhibits curiosity/passion for learning	
	Experience with research, mentoring, and/or leadership	
	Interest in pursuing biomedical/social science career	
	Demonstrates tenacity, grit, and/or academic determination	
	Fit between student’s overall life experience with BUILD EXITO goals	
	Essay 1: Influential experiences	Scoring by Site Evaluation Committee, 0–5 scale
	Essay 2: Research and career aims	
	Strength of Letters of Recommendation (2)	
Interviewing Applicants	Question 1: What challenges and barriers have you faced academically? What steps have you taken to overcome these challenges?	Site Evaluation Committee assess responses for: - Engagement - Passion for/interest in research - Passion for/interest in addressing health disparities - Academic commitment - Promise/Spark/Grit
	Question 2: What health issues have you observed or experienced in your community that have inspired you to find solutions? How do you believe that BUILD EXITO can help you address these challenges?	
Selection	Holistic evaluation of all criteria	Site Evaluation Committee

^a^Evaluation rubric uses a point system reflecting the values of the project and the considerations most important for selecting and retaining BUILD EXITO Scholars. This rubric can be modified to suit the needs of each site and applicant pool. Sites are encouraged to establish a minimum number of points needed for advancement to the interview phase of the selection process

#### EXITO scholars summer orientation

Entering EXITO scholars from all partner institutions gather at PSU for a one-week, 40-h intensive orientation that introduces the themes, goals, opportunities, and expectations associated with participation in EXITO. Scholars are supported in thinking about social determinants of health and health disparities in their own communities, and are encouraged to develop research questions aimed at examining links between these determinants and disparities. Daily research panels introduce scholars to a range of health research and opportunities available for engaging in research projects (via RLCs). Scholars hear inspiring stories about the motivations and career trajectories of researchers, with an emphasis on the careers of diverse, underrepresented scholars and faculty. Important aims for the orientation are the development of supportive bonds within the cohort and with the EXITO program, as well as the budding development of a science identity.

### EXITO curriculum

#### First-year courses

To ensure that a diverse group of students is receptive to the recruitment process described above, the EXITO project starts by laying the groundwork early. A goal of EXITO is to engage a wide cross-section of first year undergraduates in courses that introduce them to scientific concepts and methods through accessible and interesting inquiry-based and research-based instructional approaches [46, 60]. In this context, “inquiry” refers to experiential learning (e.g., an activity that requires scholars to interrogate a specific subject) assessed by direct observation, information checking, and content analysis. Research suggests that exposure to engaging classroom research experiences in early stages of undergraduate education can influence student choices to pursue research courses and careers [60]. At partner institutions, the EXITO project identifies introductory science courses and works with the instructors to incorporate research-oriented experiential learning strategies developed in conjunction with faculty affiliated with the EXITO project. At PSU, EXITO collaborates with the general education program, called University Studies, to have the broadest reach in attracting and engaging potential EXITO scholars. In these ways, EXITO facilitates broad exposure to the health sciences and generates interest in the EXITO program among first-year undergraduates, priming them to apply.

#### Gateway course for scholars

In the first year of the EXITO project (scholar sophomore status), the common curricular core for all scholars, across all partner institutions, is a Gateway course that is required prior to entering into a research placement. Such gateway courses that address the cultural norms and practices associated with science promote more inclusive involvement in undergraduate research [1]. The design of the EXITO Gateway course reflects an inquiry-based approach that incorporates multiple principles of a course-based undergraduate research experience framework [61]. The EXITO Gateway course provides a general foundation in the scientific method (e.g., modes of inquiry), research skills (e.g., conducting a literature review, framing a research question, analyzing and reporting data), and the responsible conduct of research (e.g., conflict of interest, human subjects protections). The intent of the Gateway course is socialization into research with an understanding of topics such as scientific integrity and research ethics, data acquisition and ownership, publication and peer review, laboratory safety protocols, and professional standards. Thus, the Gateway course also fulfills the NIH requirement that all training programs include instruction on the responsible conduct of research.

#### Scholar capstone

In their final year of the program (senior status), EXITO scholars conduct a student-initiated research project to apply and demonstrate acquired skills in conceiving, planning, and conducting research, yielding products for their emerging portfolios [58]. At PSU, capstone courses in the general education curriculum build cooperative learning communities by taking students out of the classroom and into the field, allowing students to integrate and apply their knowledge, skills, and interests developed over the course of their education. EXITO is working with faculty to develop new capstone opportunities to support these student-initiated projects with community partners. An alternative for EXITO Scholars at partner universities is the completion of an honors thesis or an independent-study research project. In each case, the objective is a project in which the student initiates and undertakes a self-directed project demonstrating a level of mastery that indicates preparation for graduate work.

#### Scholar workshops & seminars

EXITO offers a regular series of workshops and training seminars to support scholar success. In the first year of the program (sophomore status), enrichment workshops are designed to promote academic skill development (e.g., study skills, public speaking), encourage scientific identity development (e.g., preparing bios and personal statements), address practical concerns (e.g., financial aid, housing, child care), pursue opportunities (e.g., scholarship applications), and reflect on the socio-emotional realities of diverse students (e.g. navigating multiple contexts and identities). In the second and third years of the program (junior and senior status), the 4-year partner institutions offer scholars regular seminars on educational planning (e.g., GRE, graduate school applications), career development (e.g., preparing CV, presentation and interviewing skills, possible career paths), and research competencies (e.g., abstract submission, conference posters, manuscripts). In addition to building scholar skills and knowledge, these workshops support the building of support networks and provide valuable points of contact between scholars and EXITO program faculty.

### Scholar research experience

**Fig. 6:**
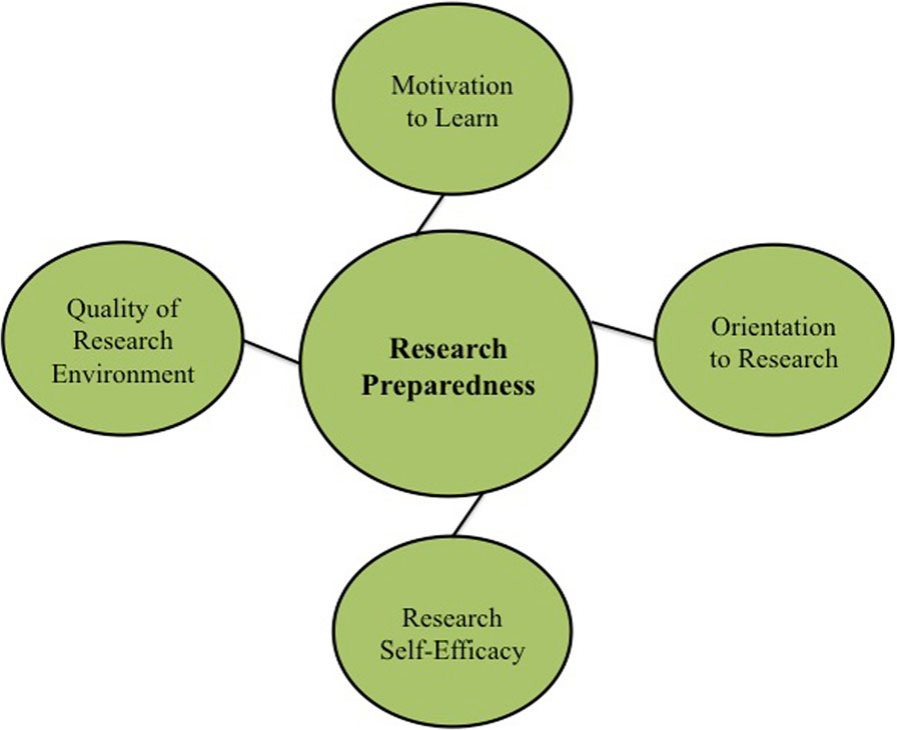
BUILD EXITO Conceptual Framework & Evaluation: Intrapersonal Level

Constructs of research preparedness frequently include: (a) Quality of research environment with specific regard to the sense of community within a research group; (b) Motivation to learn; (c) Student understanding of and feelings toward research; and (d) Student confidence in their capacity to carry out research (see Fig. 6) [58]. Consequently, the EXITO project has structured research experiences to address these constructs.

#### Research learning communities (RLCs)

Because authentic research experiences are so important for student training [62], academic persistence [46, 63], and the development of a science identity [58, 62, 64], and because research groups can offer critical social support and community learning [46], the RLCs are a centerpiece of EXITO. Research groups are designated as EXITO RLCs based on their willingness and capacity to engage undergraduate students in meaningful research activities on faculty-directed projects. As scholars become increasingly integrated into their RLCs, they have opportunities to participate in multiple facets of the research process (i.e., design, data collection, analysis, and reporting) and take ownership of their specific contributions, important aspects of becoming researchers [65]. RLCs also provide scholars with opportunities to participate in preparing proposals, writing manuscripts, giving presentations, and other scholarly activities as they gain experience and make greater contributions to the research of the team. RLC placements begin via the summer induction (described below), after completion of the Gateway course and concurrent with enrollment in a 4-year EXITO institution (rising junior status). For depth of experience, scholars are encouraged to remain in one RLC through their second and third years in the program (as junior and seniors).

#### Summer induction

The EXITO Summer Induction provides scholars with a supported entry into their RLC placements. During the preceding spring, a research fair is held to facilitate a process for matching scholars to placements. Prior to the research fair, scholars view descriptions of RLCs researchers and projects on the EXITO website to identify potential placements. During the research fair, scholars and RLC representatives meet, exchange information, and subsequently complete a brief survey to express preferences for placements. One month prior to the beginning of the new academic year (to help establish RLC routines before the start of fall classes), each EXITO scholar begins compensated work in the RLC for 16 h per week, with training, guidance, and supervision provided by a research mentor associated with the RLC. Scholars also participate in weekly four-hour seminars covering a range of relevant themes (e.g., review of responsible conduct of research, lab/research culture, effective time management). Seminars provide small-group settings in which to encourage reflection and debriefing on RLC experiences and engage in supportive peer discussion of scholar successes, challenges, and concerns. Scholars also participate in a journaling club with sessions convened by experienced researchers to help scholars develop their skills in comprehending, evaluating, and critiquing research articles [66]. As part of their compensated training awards, EXITO scholars continue to work in their RLC placements for roughly 10 h per week throughout the academic year.

#### Summer immersion

To facilitate continuation of RLC-based research between the second and third years in the program (junior to senior status), EXITO scholars engage in a compensated 10-week Summer Immersion experience. The Summer Immersion features work in the RLC as well as activities to enhance research learning and career preparation, including group seminars and journal clubs similar to those in the Summer Induction. Immersion seminars offer a more sophisticated discussion of issues based on scholars’ increased familiarity with research and provide a forum for addressing topics related to planning beyond graduation. The journal clubs help EXITO Scholars to become more proficient in interpreting and evaluating the quality of studies published in academic journals, and emphasize the mechanics of presenting research methods and findings. The experience culminates with a research symposium in which scholars present posters highlighting their RLC research. Following the Summer Immersion, scholars continue to work 10 h/week in their RLCs during their final year in the program.

## Evaluating EXITO

**Fig. 7:**
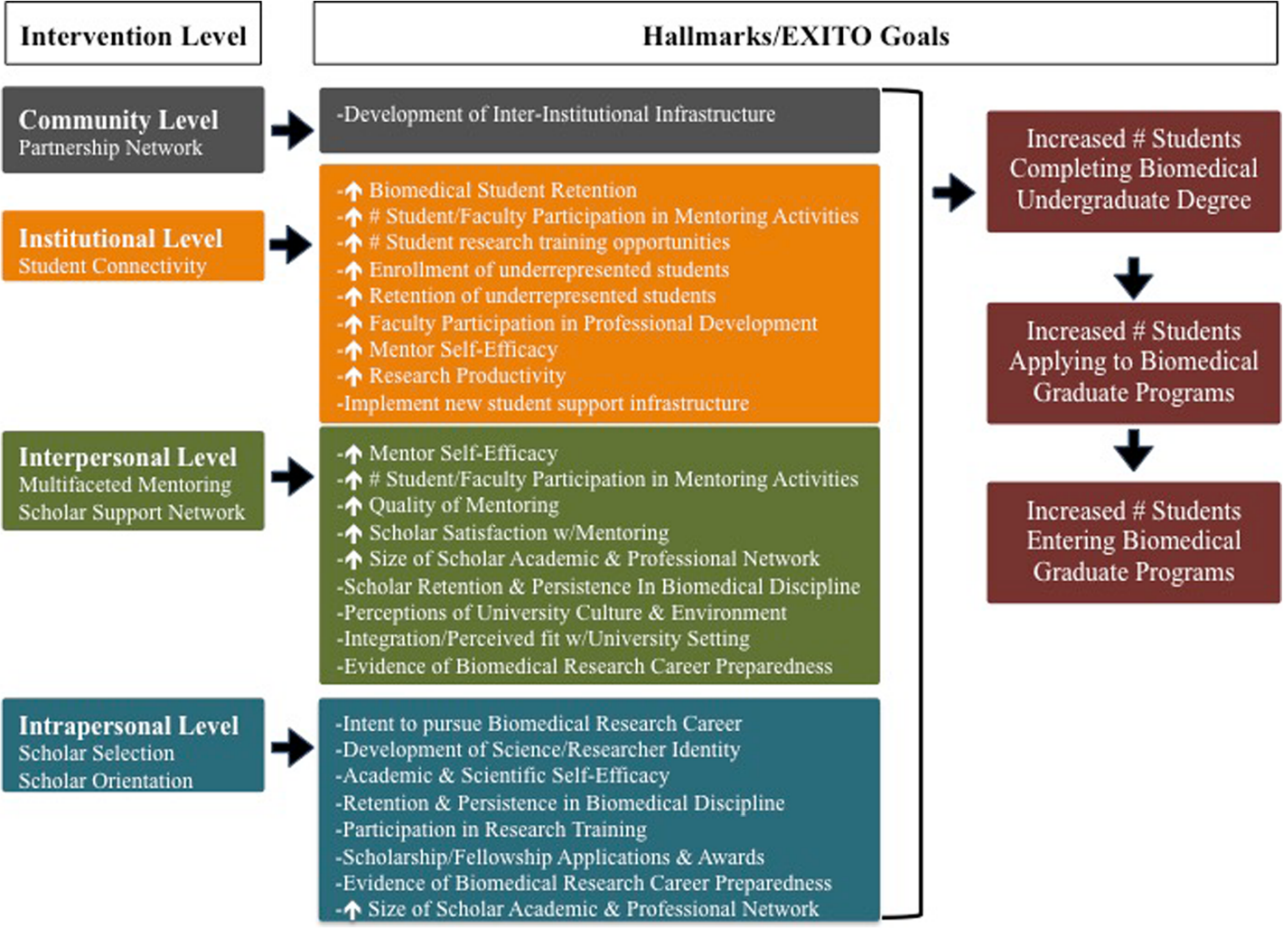
BUILD EXITO Evaluation: Hallmarks & Project Goals

Evaluation efforts combine an EXITO site-level evaluation along with the BUILD Consortium-Wide Evaluation Plan (CWEP) across all funded sites managed by the BUILD Coordination and Evaluation Center (CEC) at UCLA (see Figs. 3, 4, 5, 7). All EXITO evaluation components are designed to (a) measure the success of EXITO in meeting the BUILD hallmarks of success; (b) engage EXITO faculty and staff in ongoing process improvement of EXITO components; and (c) contribute to the body of knowledge on initiatives that further the success of underrepresented students in biomedical fields (see Fig. 7). The EXITO site evaluation uses mixed-method approaches for student, faculty, and institution level evaluation objectives. Quantitative data is collected through surveys and institutional records (IR) data, while qualitative data is collected through individual interviews and focus groups. EXITO participates in all CEC evaluation activities, including administration of the Higher Education Research Institute (HERI) surveys at Portland State University. Although the EXITO site evaluation does not use a matched comparison group of “non-treated” students, each evaluation component is designed to compare students against cohort baselines (pre/post), against selected response benchmarks on common instruments such as the HERI or PSU’s Prior Learning Survey, or against specific groups of students such as EXITO applicants or PSU science majors.

Among the BUILD sites, the EXITO project has a uniquely large number of participating partner institutions that select EXITO scholars. Aside from PSU, there are nine additional institutions with enrolled EXITO scholars. These partner institutions vary in academic calendar (semester vs. quarter), structure (two-year vs. four-year), and geography (ranging from Alaska to the Pacific Islands). These variations present significant logistical, methodological, and budgetary challenges for the EXITO evaluation, including the need to implement multiple timelines for data collection, as well as the need to connect remotely with scholars for interviews and focus groups. Identifying common measures and achieving consensus is ideal and requires substantial effort; for example, the development of a course evaluation form for the cross-EXITO Gateway Course required feedback from all partners on a format that assesses multiple modalities of course implementation. Understanding and mapping the multiple permutations of scholar pathways through EXITO, from program acceptance to graduation, is a primary objective of the EXITO site evaluation as this has implications for capturing mobility across the EXITO network.

The EXITO evaluation has resulted in new institutional infrastructure to track and assess student progress. Electronic database systems for recording and updating information on each scholar have been established for the evaluation as well as program management purposes. Unique identifiers created for each EXITO scholar and faculty member allow for consistency of data collection as students transfer among institutions. Two instruments unique to the EXITO evaluation include CRediT [67], an open-standard taxonomy for expressing roles intrinsic to research, and the Academic Support Network Assessment [68], a locally developed tool used to measure scholars’ academic networks. Originally designed to classify the diverse roles performed in the work leading to published research output, the EXITO evaluation team has expanded and adapted the CRediT tool to measure the type and level of undergraduate student participation in research labs. There are 14 roles in the taxonomy; examples include “writing” and “data curation.” EXITO scholars and their research mentors independently rate both roles and participation levels, allowing for ongoing quantitative evaluation of the degree of scholar engagement in research learning communities. The Academic Support Network Assessment is designed to measure the breadth and growth of students’ networks, with an emphasis on professional and academic networks and social capital. A baseline assessment is taken at the orientation for new scholars, and network measurements are repeated yearly until graduation.

## Challenges and opportunities

The development and initial implementation of the various EXITO project components has revealed several challenges and opportunities associated with establishing a comprehensive, multi-institutional initiative to prepare undergraduates from traditionally underrepresented populations to become biomedical researchers. Efforts to improve project performance have included conducting a broad stakeholder survey covering a variety of project management issues as well as focus groups with partner institution faculty. This section shares lessons learned at this stage of the project.

### Cross-cultural communication

A partnership network of 11 institutions spanning eight time zones presents recognizable communication challenges. Monthly partner webinars are a venue to discuss upcoming activities, make program announcements, and solicit feedback on program planning and implementation. Sub-group partner meetings are held to address the unique needs of the Pacific university, Pacific community college, and Portland-area community college partners. These meetings revealed a disconnect between “Western” approaches to communication at the EXITO primary institution and traditional norms of communication used by Pacific Island community college partners. Primary communication strategies (e.g., mass email, newsletters, large virtual meetings) focused more on *efficiency* (getting the greatest amount of information to the largest group of people with the smallest effort) than *effectiveness* (ensuring that information reached the intended groups using culturally-relevant methods that promote understanding, engagement, and action). Thus, resources have been dedicated to visit Pacific Island campuses to provide face-to-face training and support, observe changes in institutional infrastructure, and to connect with key administrators.

### Top-down and bottom-up planning and implementation

As noted, the institutional structure of the BUILD program calls for a primary institution (PSU), a research partner (OHSU), and pipeline partners (2-year community colleges). This structure lends itself to top down planning where key decisions are made by the primary and research intensive institutions. This structure makes sense with some BUILD components, such as institutional development, where the expertise at OHSU can guide the partnership for building a research portfolio and promoting faculty development. However, this structure does not lend itself to planning and implementation for curriculum development, recruitment, scholar training and enrichment, and academic support. Top-down approaches can overlook and overshadow the interests and expertise of faculty at the pipeline institutions, diminishing their sense of shared responsibility for program planning and implementation. Moreover, grassroots leaders often identify problems and act to implement solutions in daily work that begin to change the culture of the institution [69]. The annual Curriculum Development Conference addresses some of these planning challenges by providing opportunities for shared expertise and responsibility for curriculum planning. As another strategy for bottom-up planning, EXITO-wide working groups comprised of faculty from each institution work together on activities like recruitment, scholar selection, and student support.

### Flexibility with scholar program milestones

The EXITO project involves a series of milestones that scholars meet sequentially, from freshman to senior year. While this sequencing works for most, typical barriers to access and completion of academic programs (family obligations, part-time enrollment, delays in academic progress) challenge some scholars in meeting target learning objectives. These challenges require processes and procedures for flexible completion of program requirements in ways that respond to the unique collection of challenges that each scholar faces, while not compromising the learning outcomes and aims of the project. The Student Success Committee assists scholars with accommodations and support plans when they are struggling to meet program expectations.

## Conclusion

The EXITO project provides comprehensive training opportunities for hundreds of aspiring researchers from traditionally underrepresented student populations enrolled across a large network of eleven institutions in U.S. Pacific states and territories. Shaped by a theoretical framework emphasizing a spectrum of intervention approaches, EXITO includes specific activities across multiple levels to engage, retain, and train students in science, provide an enriched science and career development curriculum, place scholars in meaningful long-term research experiences, connect scholars with multiple mentors, and create a supportive community through social connections and services. This innovative, three-year scholar training model is designed to accommodate the large number of diverse transfer students from community college partners that enroll at the primary institution, with the full model replicated at our 4-year partner institutions. The project also focuses on institutional development to ensure sustainable success at each level of the model, supporting faculty with curriculum development, research capacity building, and mentor training. All of these transformational efforts are rigorously evaluated, with stakeholder surveys and focus groups conducted to identify areas for program improvement. For the faculty and staff who are members of the EXITO team across the partnership network, the rewards of this work have been experienced already—whenever we witness the enthusiasm, achievement, and appreciation of tremendous scholars striving to fulfill their boundless potential.

## Abbreviations

BUILD: Building University Infrastructure Leading to Diversity
CEC: BUILD Coordination and Evaluation Center
CWEP: BUILD Consortium-Wide Evaluation Plan
EXITO: Enhancing Cross-disciplinary Infrastructure and Training at Oregon
NIH: National Institutes of Health
OHSU: Oregon Health & Science University
PRP: Pilot Research Program
PSU: Portland State University
RLC: Research Learning Community
